# Diagnostic accuracy of ultrasonography in the assessment of anterior knee pain

**DOI:** 10.1186/s13244-020-00914-2

**Published:** 2020-10-01

**Authors:** Mohammad Abd Alkhalik Basha, Diaa Bakry Eldib, Sameh Abdelaziz Aly, Taghreed M. Azmy, Nader E. M. Mahmoud, Tarek Mohamed Ghandour, Tarek Aly, Shimaa Mostafa, Asmaa M. Elaidy, Hesham Youssef Algazzar

**Affiliations:** 1 Department of Radiodiagnosis, Faculty of Human Medicine, Zagazig University, Zagazig, Egypt; 2 Department of Radiodiagnosis, Faculty of Human Medicine, Benha University, Benha, Egypt; 3 Department of Orthopaedic Surgery, Faculty of Human Medicine, Ain Shams University, Cairo, Egypt; 4 Department of Orthopaedic Surgery, Faculty of Human Medicine, Tanta University, Tanta, Egypt; 5 Department of Rheumatology and Rehabilitation, Faculty of Human Medicine, Zagazig University, Zagazig, Egypt; 6 Department of Psychiatry, Faculty of Human Medicine for Girls, Al-Azhar University, Zagazig, Egypt

**Keywords:** Knee joint, Ultrasonography, Magnetic resonance imaging, Anterior knee pain, Diagnostic accuracy

## Abstract

**Background:**

Anterior knee pain (AKP) is a problematic complaint, considered to be the most frequent cause of orthopedic consultancy for knee problems. This study aimed to highlight diagnostic accuracy of ultrasonography as a fast imaging technique in assessment of patients with AKP.

**Methods and results:**

A prospective study was conducted on 143 patients with clinically confirmed AKP. All patients underwent ultrasonography and MRI examinations of the knee. The diagnostic accuracy of ultrasonography compared to MRI for evaluating different findings of possible causes of AKP were analyzed using receiver operating characteristic (ROC) curve and judged by area under curve (AUC). A total of 155 knees were included in the study; 26 knees showed no abnormalities, 19 knees showed positive MRI only, and 110 knees showed positive ultrasonography and MRI. Ultrasonography and MRI reported 11 different findings of possible causes of AKP or related to it. Joint effusion was the most common finding (38%) followed by trochlear cartilage defect (20.6%) and superficial infrapatellar subcutaneous edema (20%). The overall accuracy of ultrasonography was 85.3% sensitivity and 100% specificity. The ultrasonography provided the highest sensitivity (100%) in detecting bipartite patella, followed by 91.5% for joint effusion, and 87.5% for quadriceps tendinopathy. The ROC curve analysis of overall accuracy of ultrasonography showed an AUC of 0.93. The overall Kappa agreement between ultrasonography and MRI was good (*k* = 0.66).

**Conclusion:**

Ultrasonography can be used to make a swift screening and assessment of painful anterior knee and as an alternative to MRI when it is unavailable or contraindicated.

## Key points

- Ultrasonography showed high diagnostic accuracy in detecting most causes of AKP.
- Although MRI is the gold standard technique for AKP imaging, ultrasonography can be used to make a swift screening and assessment of the painful anterior knee and can be used as an alternative to MRI when MRI is unavailable or contraindicated.
- MRI may be warranted if a patellar cartilage defect is clinically suspected or the ultrasonography yielded negative results.

## Background

Anterior knee pain (AKP) is a problematic complaint, considered to be the most frequent cause of orthopedic consultancy for knee problems [1]. AKP’s differential diagnosis falls within a wide range, which is still overlapping and lacking clear understanding [2]. It is more frequent in young people between 15 and 30 years and is more frequent in women than in men. Despite its prevalence, AKP nature and causes remain inadequately understood and can be bothersome for patients and clinicians as it causes chronic disability, limited sports and activity, and a negative impact on the quality of life [3, 4]. Many authors linked AKP to the patellofemoral pathology, especially the patellofemoral instability, while others clearly reported that structural anomalies did not provide a complete explanation of the pain [5]. Moreover, structural anomalies were found to be minor among AKP patients, and there was no obvious correlation between patellofemoral malalignment and long-term results of AKP treatment [6].

The patient’s clinical history and physical examination are of paramount importance in diagnosing the cause of AKP. The physical examination is complemented by imaging examinations, and the combination should yield a precise diagnosis that will be the cornerstone in developing an appropriate therapeutic program [7]. Imaging workup is important to evaluate the extent of the bone and soft tissue abnormality and guide therapeutic intervention if needed [8, 9]. The most helpful diagnostic techniques for evaluating soft tissue changes are ultrasonography and magnetic resonance imaging (MRI). Plain radiography is of limited value, and computed tomography is not recommended [10, 11]. Most orthopedics rely on MRI as the method of choice in knee imaging as it provides high contrast resolution images not only for the soft tissue but also for the underlying bone and allowing a precise assessment of the underlying cause [3, 12]. Moreover, MRI has replaced diagnostic arthroscopy as the primary diagnostic modality for many knee pathologies [13]. Ultrasonography has become more popular because it is safe, quick, inexpensive, and reliable. It has the ability to assess soft tissues in the anterior aspect of the knee, which could be the main source of pain [6].

Although the literature is full of several researches about the value of ultrasonography examination of the knee, the AKP dilemma makes the need for evidence-based value, regarding various diagnoses, useful for guiding choices in value-based health care in imaging, a point that should be stressed. Therefore, we conducted our prospective study to clarify the diagnostic accuracy of ultrasonography as a fast imaging technique in the assessment of patients with AKP and comparing the results with MRI.

## Methods

### Ethical statement

Ethical approval was obtained from the local research ethical committee. All patients were informed of the study and provided written informed consent prior to ultrasound and MRI examination. The study was performed in accordance with the ethical principles of the Declaration of Helsinki.

### Study population

**Fig. 1:**
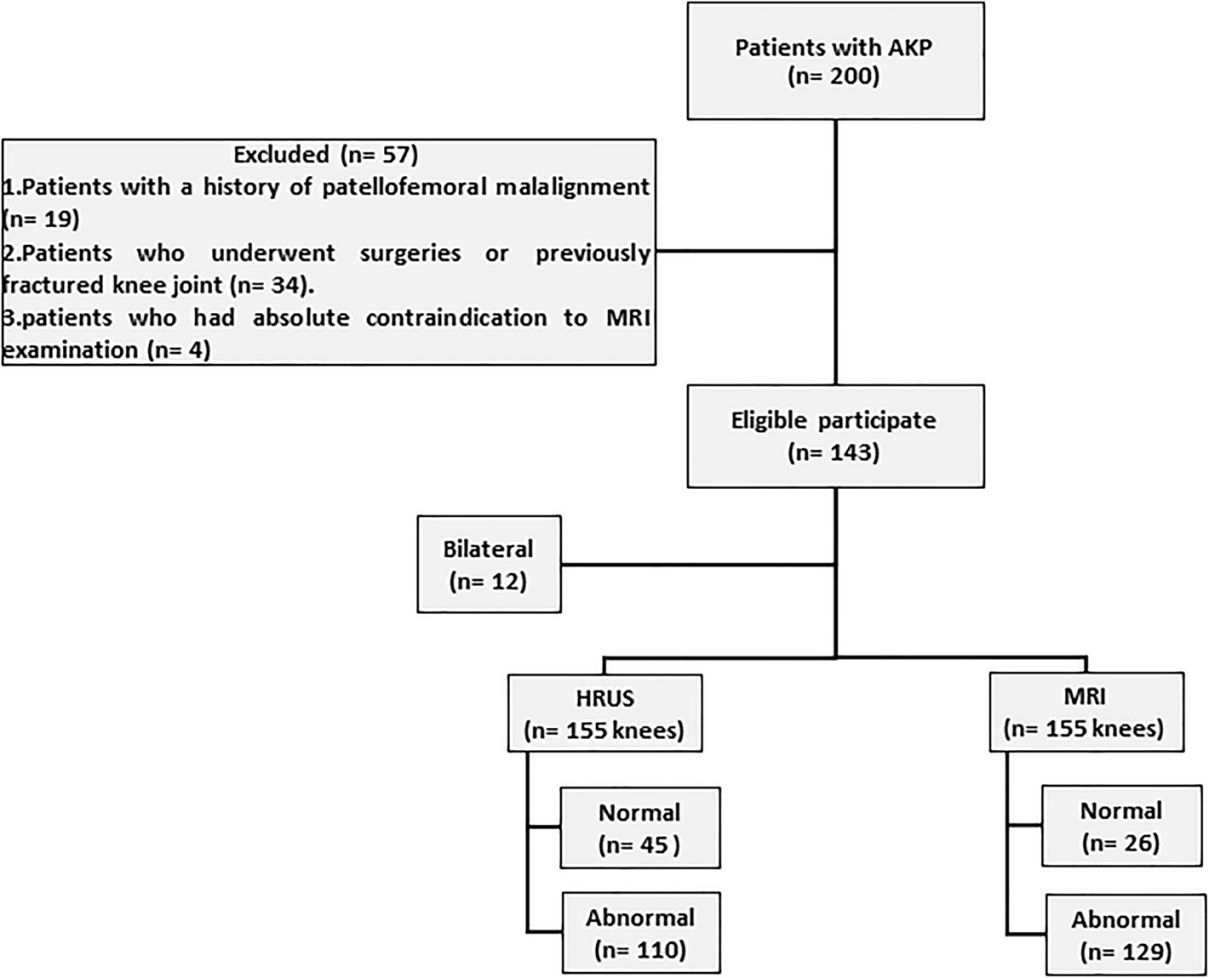
Flow chart of our study. AKP, anterior knee pain; n, number; MRI, magnetic resonance imaging

This prospective study was carried out between August 2018 and January 2020. Inclusion criteria were patients with a clinically confirmed AKP and scheduled for MRI examination of the knee. Initially, we collected 200 consecutive patients. Exclusion criteria were (i) patients with a history of patellofemoral malalignment since the ultrasonography diagnostic criteria for patellofemoral malalignment is not established yet (*n* = 19), (ii) patients who underwent surgeries or previously fractured knee joint (*n* = 34), and (iii) patients who had absolute contraindications to MRI examination (*n* = 4). The exclusion process resulted in a final cohort comprised of 143 patients. Fig. 1 illustrates the flow chart of our study.

### Ultrasonography and color Doppler technique and analysis

Gray-scale and color Doppler ultrasonography of the knee were performed using Aplio 400, Toshiba ultrasound scanner, with a high-resolution, multifrequency linear transducer (7–12 MHz). Patients were positioned in a supine position with the knee comfortably flexed (30–45°) by placing a pillow under the knee. To avoid loss of contact, we used plenty of thick gel. The exam started at the suprapatellar region by scanning in the long axis plane from medial to lateral. The quadriceps tendon was scanned first in both long axis and short axis planes. The trochlear cartilage, as well as the medial and lateral patellar recesses, was examined in various degrees of knee flexions. Long and short axis planes for the patellar tendon were then obtained. The parameters of the color Doppler mode was set to depict the slow flows by using high Doppler frequency, low pulse repetition frequency, minimal wall filter, and high color gain. Focus is positioned just deep to the area of interest. Any suspected lesion, firmness, or tenderness was examined by moving the probe over and around the lesion [14–16]. Dynamic ultrasonography examination executed by changing the degree of knee flexion as well as by medial and lateral movements of the patella was also done. During the ultrasonography examination, the knee was divided into the following entities: (i) the extensor mechanism, i.e., quadriceps tendon, patella, and patellar tendon; (ii) the trochlear femoral articular cartilage; (iii) anterior knee joint recesses (suprapatellar and both medial and lateral recesses); (iv) anterior knee bursa, i.e., subcutaneous prepatellar bursa, subcutaneous infrapatellar bursa, and deep infrapatellar bursa; (v) suprapatellar fat and deep infrapatellar Hoffa’s fat pad; and (vi) miscellaneous causes.

**Fig. 2:**
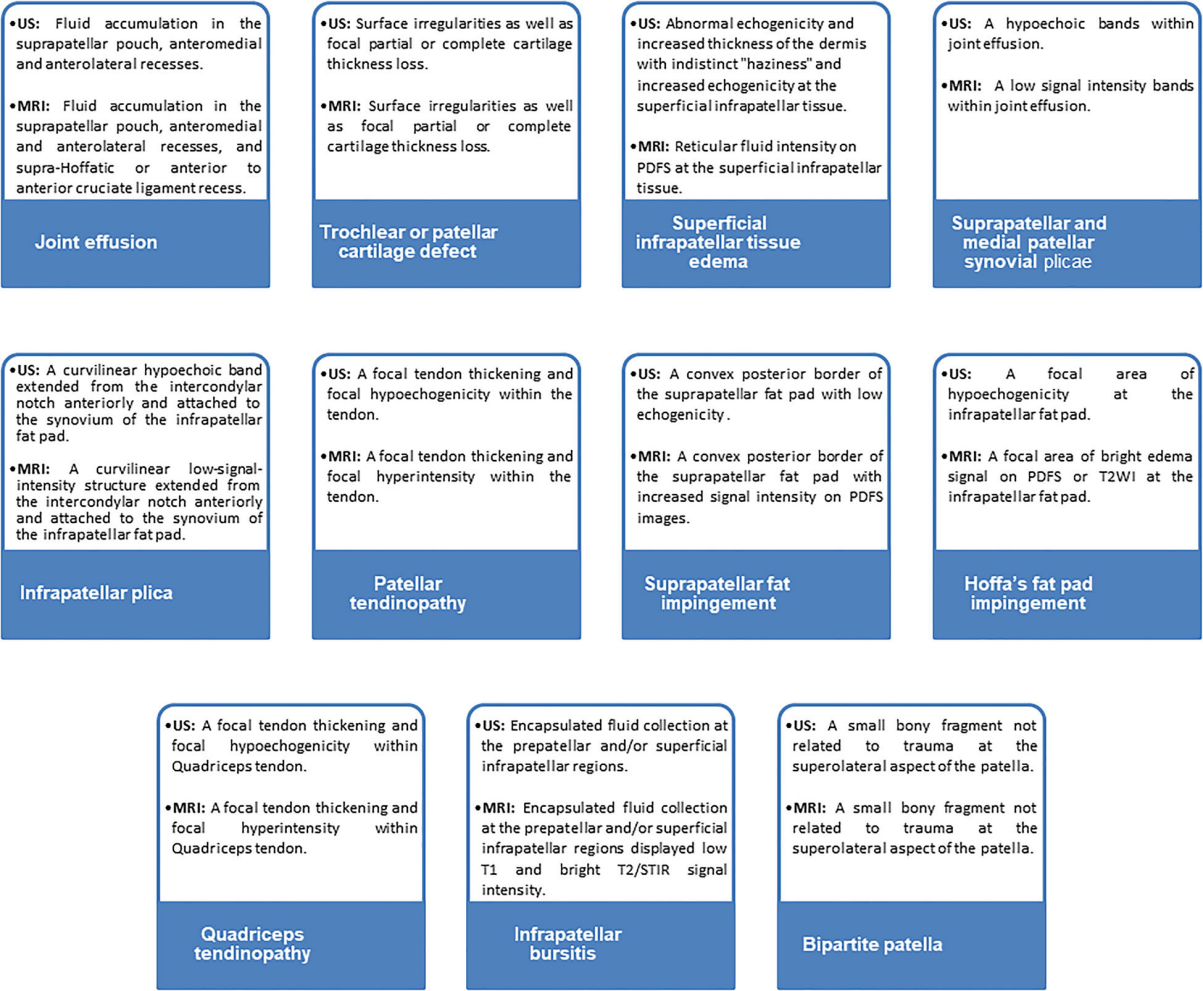
Ultrasonography and MRI findings of AKP. US, ultrasonography; MRI, magnetic resonance imaging; AKP, anterior knee pain; PDFS, proton density fat sat; STIR, short tau inversion recovery

All ultrasonography examinations were performed by one highly experienced musculoskeletal radiologist (with over 10 years of musculoskeletal ultrasound experience and had performed > 1000 ultrasound examinations per year). The radiologist was blinded to clinical data. Ultrasonography findings of AKP are summarized in Fig. 2. Hypervascularity on color Doppler examinations was used as a marker for patellar tendinopathy, Hoffa’s or suprapatellar fat pad impingement, and inflammation.

### MRI protocol and image analysis

MRI was performed during a timeframe of 1 week after the ultrasonography. The applied MRI protocol was revised to ensure its compliance with the study requirements. MRI was performed using 1.5-T Toshiba Vantage Elan System. A Flex Speeder 16 channel (receiver only) coil was used. The MRI protocol consisted of fat-suppressed and non-fat-suppressed sequences. The protocol for the evaluation of the knee is listed in Table 1.

**Table 1:** MRI protocol for evaluation of the knee

	TR (ms)	TE (ms)	Thickness (mm)	Interslice gap (%)	FOV (cm)	Matrix	Scan time (min)
T2 FSE axial	3200–3800	110	3	10	17 × 17	356 × 286	3.25
PDFS axial	2100–2600	36	3	10	16 × 16	284 × 256	3.40
PDFS sagittal	2100–2600	36	3	10	16 × 16	284 × 256	3.04
T1 FSE sagittal	400–410	10	3	10	16 × 16	56 × 286	2.5
PDFS coronal	2100–2600	36	3	10	16 × 16	284 × 256	2.45

*FSE* fast spine echo, *PDFS* proton density fat sat, *TR* repetition time, *TE* echo time, *FOV* field of view, *MRI* magnetic resonance imaging, *ms* millisecond, *cm* centimeter, *min* minutes

All MRI data were transferred to the workstations, and image analysis was performed on the PACS system (PaxeraUltima—paxeramed). All MRI images were interpreted by one radiologist with over 17 years of experience in musculoskeletal imaging. The radiologist was blinded to the clinical history and ultrasound findings. MRI findings of AKP are summarized in Fig. 2.

### Statistical analysis

Demographic data are presented as frequency and percentages. Diagnostic accuracy of ultrasonography included sensitivity, specificity, positive predictive values (PPV), and negative predictive value (NPV) as compared to MRI for evaluating different anterior knee findings were analyzed using receiver operating characteristic curve (ROC) and judged by the area under the curve (AUC). The diagnostic significance of the determined AUC was compared to the null hypothesis that AUC = 0.5, which is the area under the diagonal reference line. We use the method of Delong et al. [17] for the calculation of the standard error of the AUC. Cohen’s *κ* was run to determine if there was an agreement between the ultrasonography and MRI on identifying different lesions in patients’ knees. Interpretation of *κ*-statistic value is based on Altman [18] as moderate agreement (*κ* = 0.41–0.6), good agreement (*κ* = 0.61–0.80), and very good agreement (*κ* = 0.81–1). Statistical analysis was conducted using Medcalc software version 19.1, 2019, and IBM SPSS statistics version 26, 2019, for Windows statistical package. A *p* value ≤ 0.05 is considered statistically significant.

## Results

### Patients

A total of 155 knees from 143 patients (80 males and 63 females; mean age, 33.6 ± 13.9 years; range, 12–62 years) were included in our study. The most common age group was between 26 and 35-year-old (30%). Twelve patients (8.4%) had bilateral AKP. The chief complaint in our patients was knee pain or disability, not more than 6 months.

### Ultrasonography and MRI findings

**Fig. 3:**
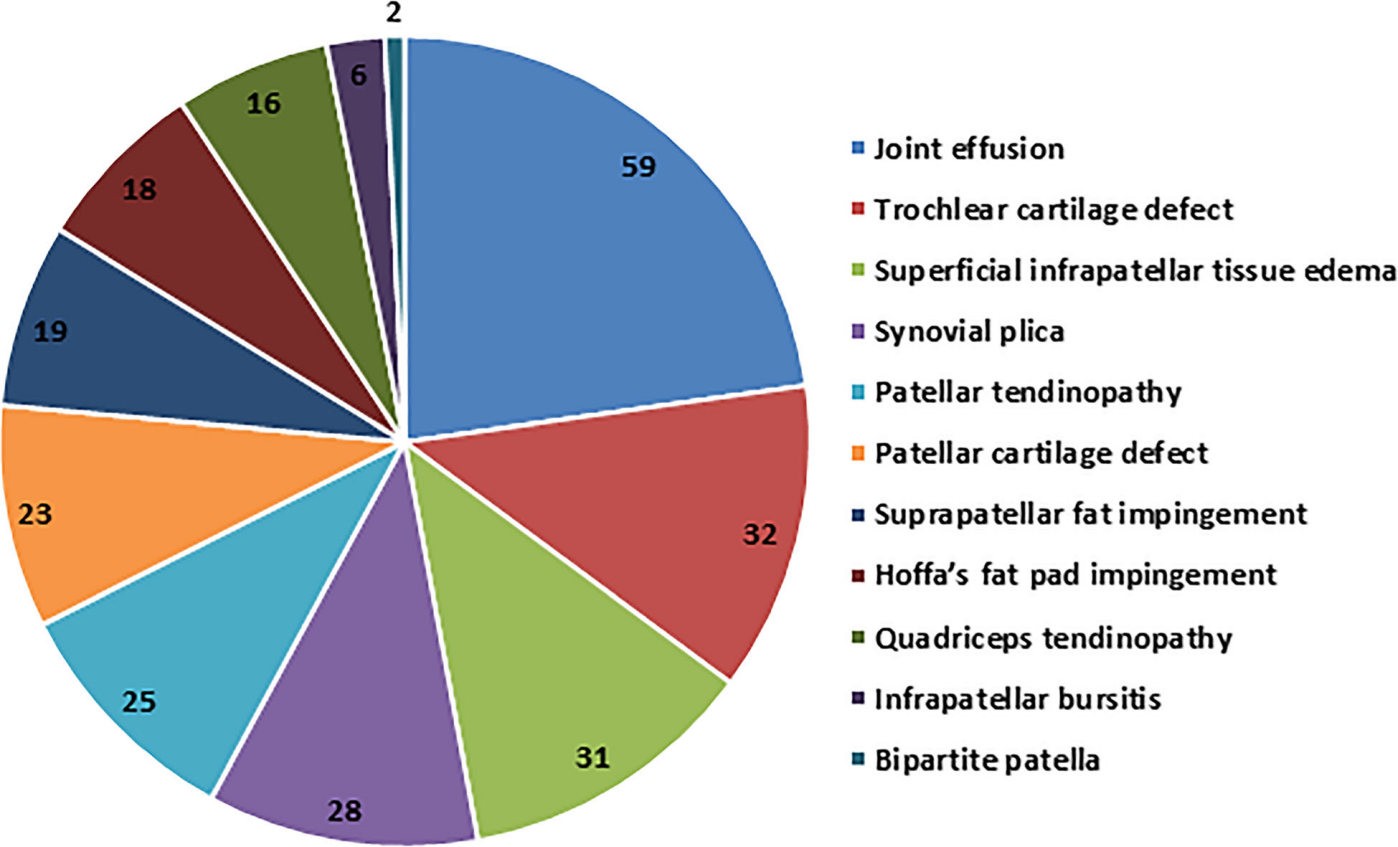
Frequency of AKP findings as detected by MRI. AKP, anterior knee pain; MRI, magnetic resonance imaging

The ultrasonography detected 195 findings in 110 knees, compared to 259 findings in 129 knees detected by MRI. Ultrasonography revealed 45 knees with no findings compared to 26 knees by MRI. The number of findings per technique is shown in Table 2. Both ultrasonography and MRI reported 11 different findings of possible causes of AKP or related to it (Table 3). Joint effusion was the most common finding (59 patients, 38.1%), while trochlear cartilage defect and superficial infrapatellar subcutaneous edema were reported in 20.6% and 20%, respectively (Fig. 3). Ultrasonography was not able to detect any of 23 knees with patellar cartilage defect and detected 23 out of 32 knees with trochlear cartilage defects. MRI showed 28 knees with evidence of plica: 13 suprapatellar plicae, 10 medial plicae, and 5 infrapatellar plicae. Ultrasonography detected 12 out of the 13 suprapatellar plicae and all medial patellar plicae. Ultrasonography was not able to detect the infrapatellar plicae. Ultrasonography detected 21 out of 25 knees with patellar tendinopathy, 14 out of 16 knees with quadriceps tendinopathy, and 16 out of 19 knees with suprapatellar pad of fat impingement. MRI showed 18 knees with Hoffa’s fat pad impingement; 12 were identified by ultrasonography, and 6 were missed. Three out of 12 knees showed increase vascularity on color Doppler examination. Ultrasonography detected the two knees with bipartite patella and 4 out of 6 knees with infrapatellar bursitis.

**Table 2:** Number of findings in each knee as detected by ultrasonography and MRI

Number of findings	Ultrasonography	MRI
No findings	45	26
One finding	49	52
Two findings	42	44
Three findings	14	17
Four findings	5	12
Five findings	0	4

The data represent the number of affected knees

*MRI* magnetic resonance imaging

**Table 3:** Frequency of each finding as detected by ultrasonography

	TP	FP	FN	TN
Joint effusion	54	4	5	92
Trochlear cartilage defect	23	2	9	121
Superficial infrapatellar tissue edema	24	0	7	124
Synovial plica	22	0	6	127
Patellar tendinopathy	21	0	4	130
Patellar cartilage defect	0	0	23	132
Suprapatellar fat impingement	16	0	3	136
Hoffa’s fat pad impingement	12	0	6	137
Quadriceps tendinopathy	14	0	2	139
Infrapatellar bursitis	4	0	2	149
Bipartite patella	2	0	0	153

*TR* true positive, *FP* false positive, *FN* false negative, *TN* true negative

### Diagnostic accuracy of ultrasonography

Out of the 155 knees, 26 (16.8%) showed no abnormalities by both ultrasonography and MRI, 110 (71%) showed positive findings by both ultrasonography and MRI, and 19 (12.2%) showed positive findings by MRI, whereas ultrasonography was reported to be normal. The overall diagnostic accuracy of ultrasonography in detecting the abnormal findings in AKP patients was 85.3% sensitivity, 100% specificity, 100% PPV, and 57.8% NPP. The ultrasonography provided the highest sensitivity (100%) in detecting the bipartite patella, followed by 91.5% for joint effusion, 87.5% for quadriceps tendinopathy, 84.2% for suprapatellar fat impingement, and 84% for patellar tendinopathy (Table 4).

**Table 4:** Diagnostic accuracy of ultrasonography findings using MRI as the gold reference standard

	Sensitivity (%)	Specificity (%)	PPV	NPV	AUC (ROC)	Kappa agreement (Cohen’s Kappa)
Joint effusion	91.5	95.8	93.1	94.8	0.93***	0.87***
Trochlear cartilage defect	71.9	98.4	92	93.1	0.85***	0.76***
Superficial infrapatellar tissue edema	77.4	100	100	94.7	0.88***	0.83***
Synovial plica	78.6	100	100	95.5	0.88***	0.84***
Patellar tendinopathy	84	100	100	97	0.92***	0.89***
Patellar cartilage defect	0	100	-	85.2	0.5^#^	0^¥^
Suprapatellar fat impingement	84.2	100	100	97.8	0.92***	0.9***
Hoffa’s fat pad impingement	66.7	100	100	95.8	0.83***	0.775***
Quadriceps tendinopathy	87.5	100	100	98.6	0.94***	0.93***
Infrapatellar bursitis	66.7	100	100	98.4	0.83^¥¥^	0.79***
Bipartite patella	100	100	100	100	1***	1***
Overall validity	85.3	100	100	57.8	0.93***	0.66***

*MRI* magnetic resonance imaging, *PPV* positive predictive value, *NPV* negative predictive value, *CI* confidence interval, *AUC* area under curve, *ROC* receiver operating characteristic curve

***p value < 0.001

^#^*p* value > 0.05 (non-significant)

^¥^No *p* value can be calculated

^¥¥^*p* value = 0.002

### ROC curve analysis

**Fig. 4:**
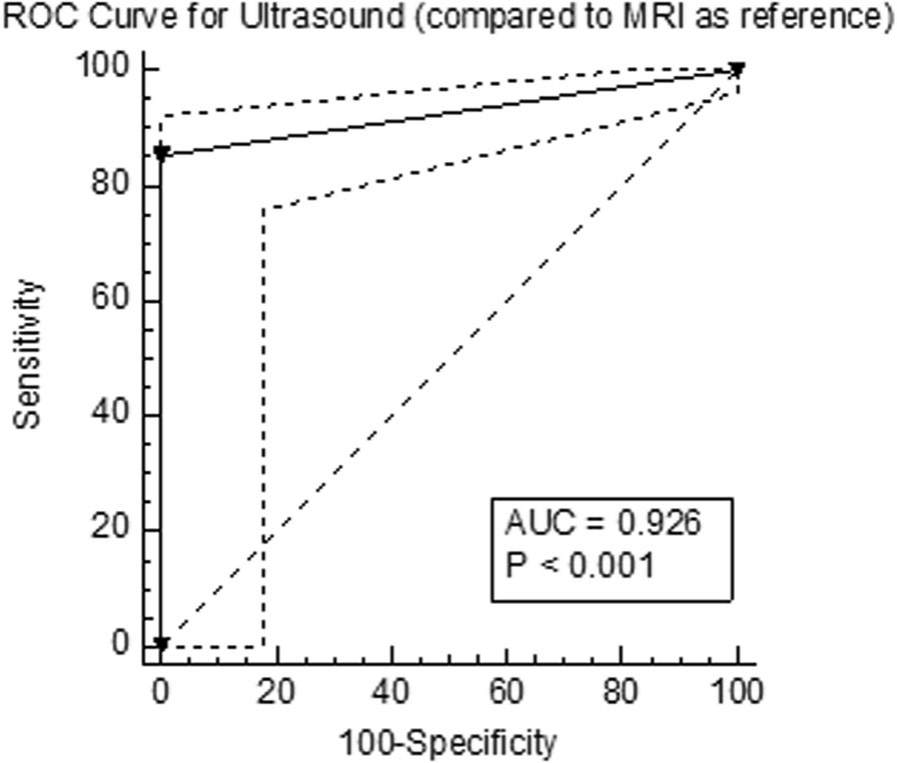
ROC curve analysis of ultrasonography for the identification of anterior knee findings compared to MRI. ROC, receiver operating characteristic curve; MRI, magnetic resonance imaging; AUC, area under the curve

Table 4 summarizes the ROC curve and the Cohen Kappa analysis for each finding. The ROC curve analysis of the diagnostic accuracy of ultrasonography for detecting the abnormal findings in AKP patients in comparison to MRI showed that the highest diagnostic accuracy was in detecting quadriceps tendinopathy (AUC = 0.94, *p* < 0.001), followed by joint effusion (AUC = 0.93, *p* < 0.001), and impingement of suprapatellar fat (AUC = 0.92, *p* < 0.001). The ROC curve analysis of the overall diagnostic accuracy of ultrasonography showed an AUC of 0.93 (95% CI = 0.72–0.91, *p* < 0.001) (Fig. 4). The overall Kappa agreement between ultrasonography and MRI was good (*k* = 0.66, 95% CI = 0.53–0.80).

**Fig. 5:**
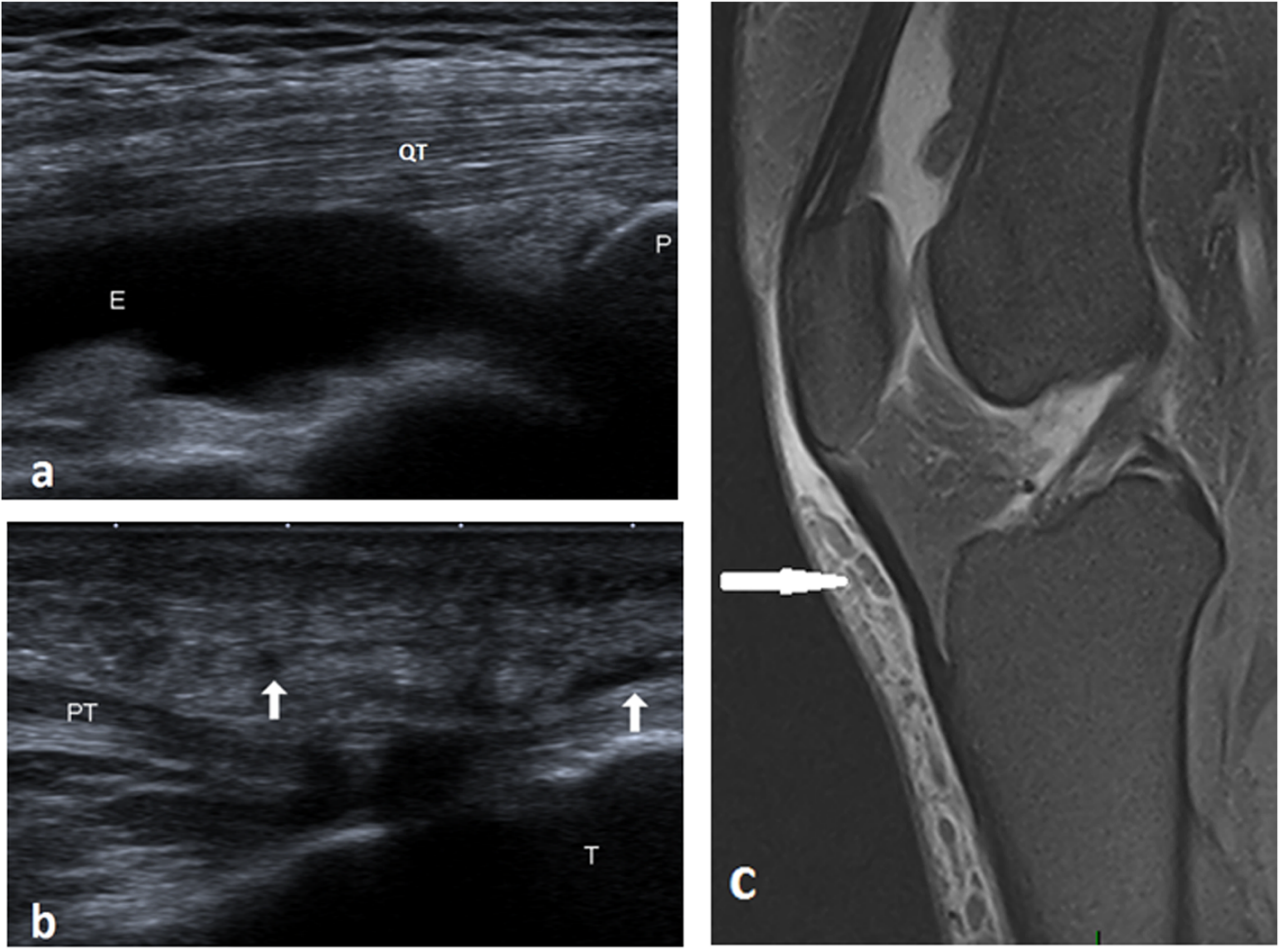
A 44-year-old male with joint edema and anterior subcutaneous edema. **a** Long axis ultrasound image illustrates joint effusion in the suprapatellar pouch. **b** Long axis ultrasound scan illustrates hypoechoic subcutaneous reticulations anterior to the patellar tendon (arrows). **c** Sagittal PDFS MRI illustrates joint effusion, prepatellar, and superficial infrapatellar reticular fluid intensities of edema. QT, quadriceps tendon; PT, patellar tendon; E, effusion; P, patella; T, tibia

**Fig. 6:**
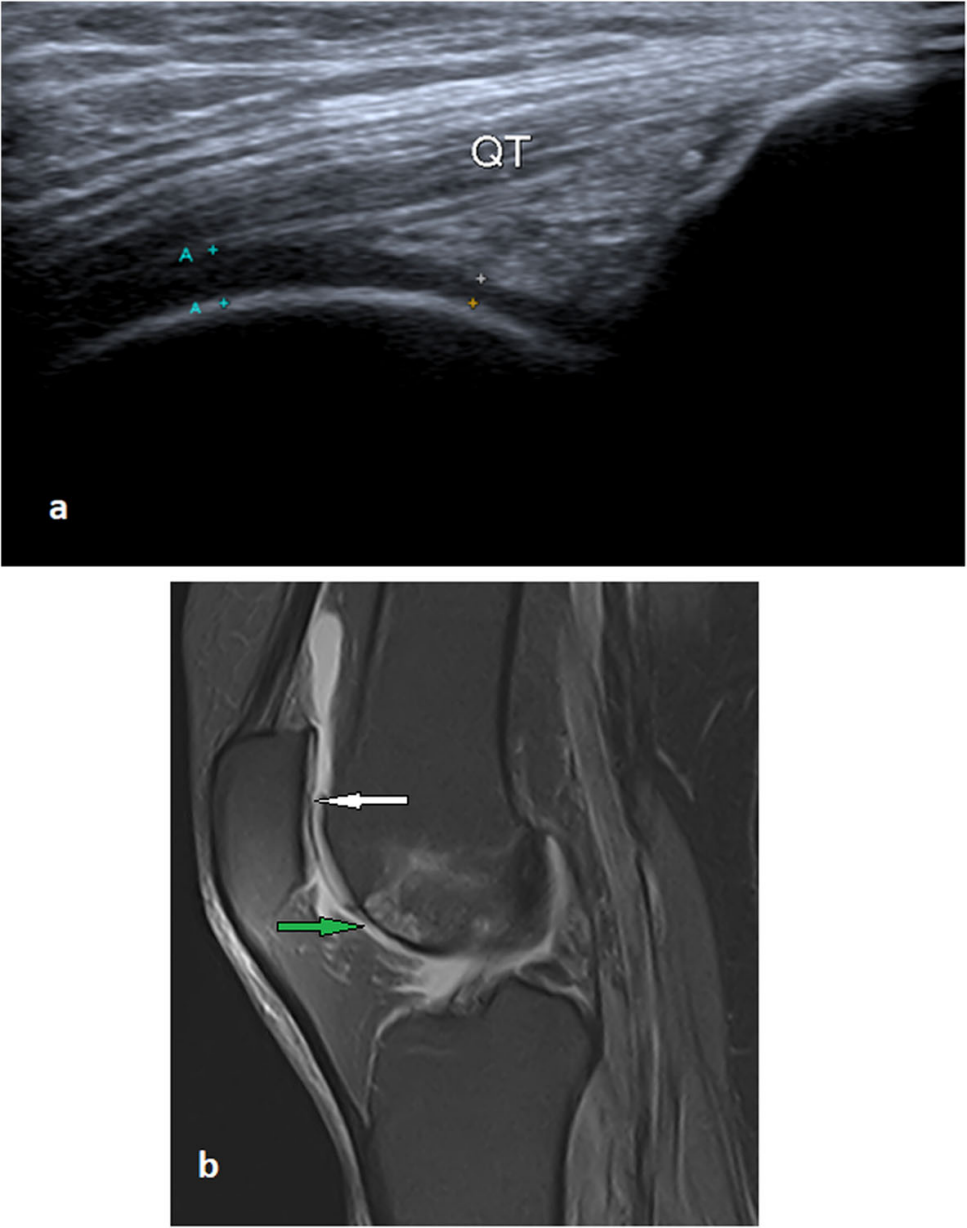
A 54-year-old male with focal patellar and trochlear partial thickness cartilage loss, mild joint effusion, and anterior infrapatellar subcutaneous edema of the left knee. **a** Long axis ultrasound image of the left knee illustrates a focal thinning of the anteroinferior aspect of the trochlear cartilage. **b** Sagittal PDFS MRI illustrates partial thickness cartilage loss of the patella (white arrow), partial-thickness cartilage loss of the anteroinferior surface of the trochlear cartilage with subchondral bone marrow changes (green arrow), joint effusion, and anterior subcutaneous edema. QT, quadriceps tendon

**Fig. 7:**
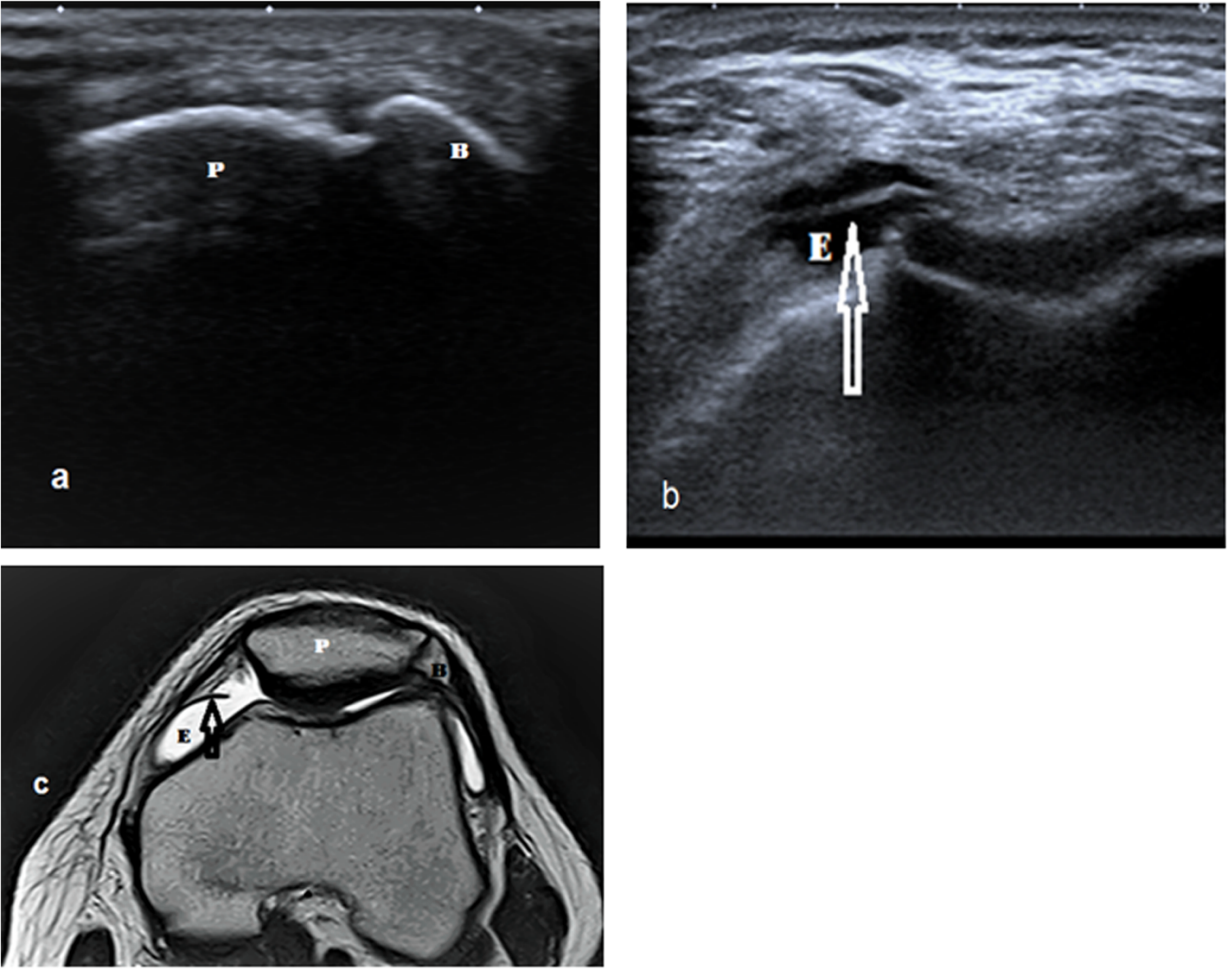
A 31-year-old male with a bipartite patella, joint effusion, and medial patellar plica. **a** Short axis ultrasound image illustrates the cleft between the patella and bipartite fragment at the superolateral pole of the patella. **b** Short axis ultrasound image illustrates joint effusion and medial patellar plica (arrow). **c** Axial T2WI MRI illustrates bipartite patella, joint effusion, and medial patellar plica. P, patella; E, effusion; B, bipartite patellar fragment

**Fig. 8:**
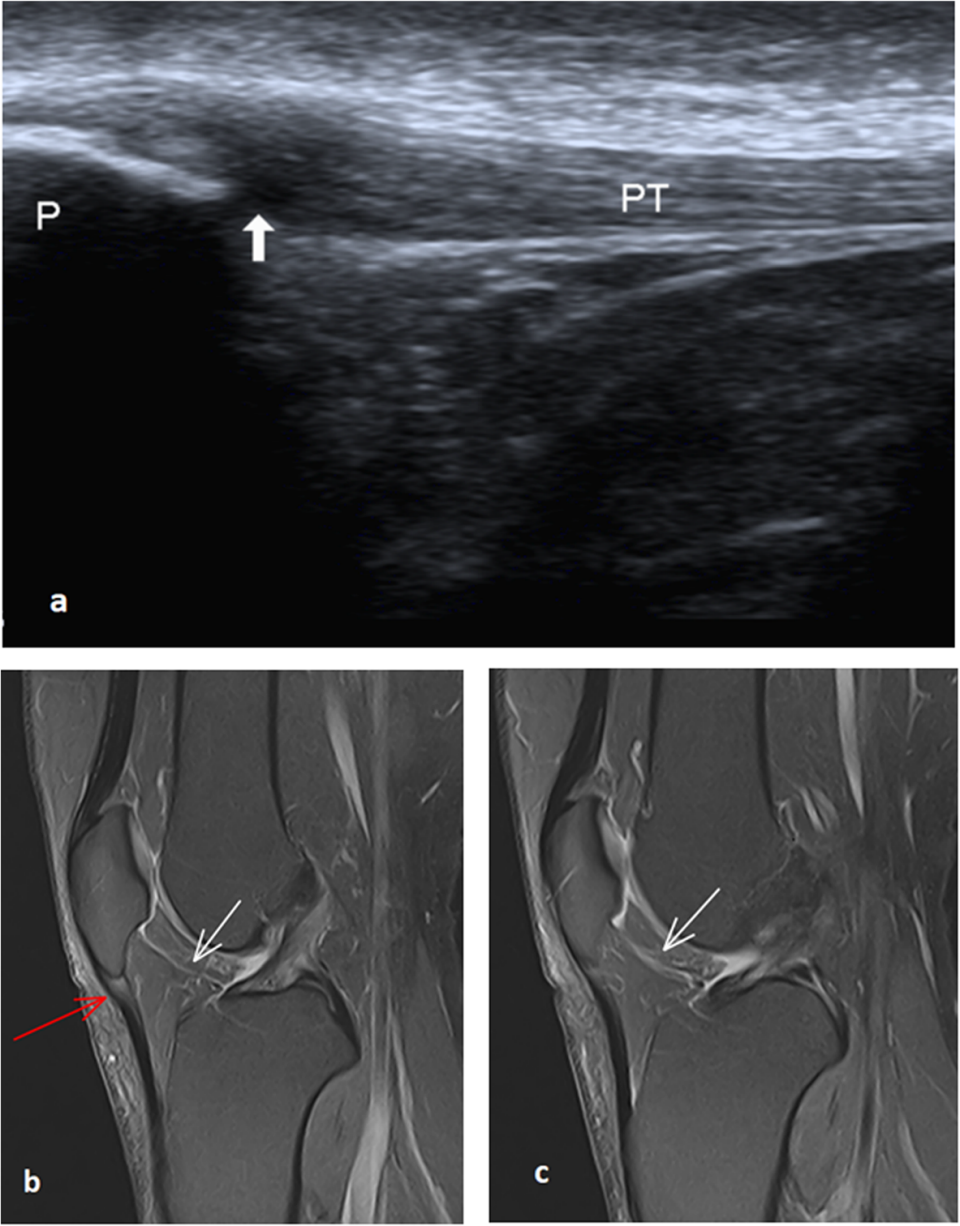
A 45-year-old female with right patellar tendinopathy and infrapatellar plica. **a** Long axis ultrasound image through the patellar tendon shows a focal thickening of the proximal part of the patellar tendon with a localized hypoechoic area sparing the anterior fibers. (White thick arrow). **b** and **c** Two subsequent sagittal PDFS MRI images through intercondylar notch show focal proximal tendon thickening and increased signal intensity (red arrow) sparing the anterior tendon fibers. Infrapatellar plica (white arrow) appears as a curvilinear high signal passing through Hoffa’s fat pad (not detected by ultrasound). P, patella; PT, patellar tendon

**Fig. 9:**
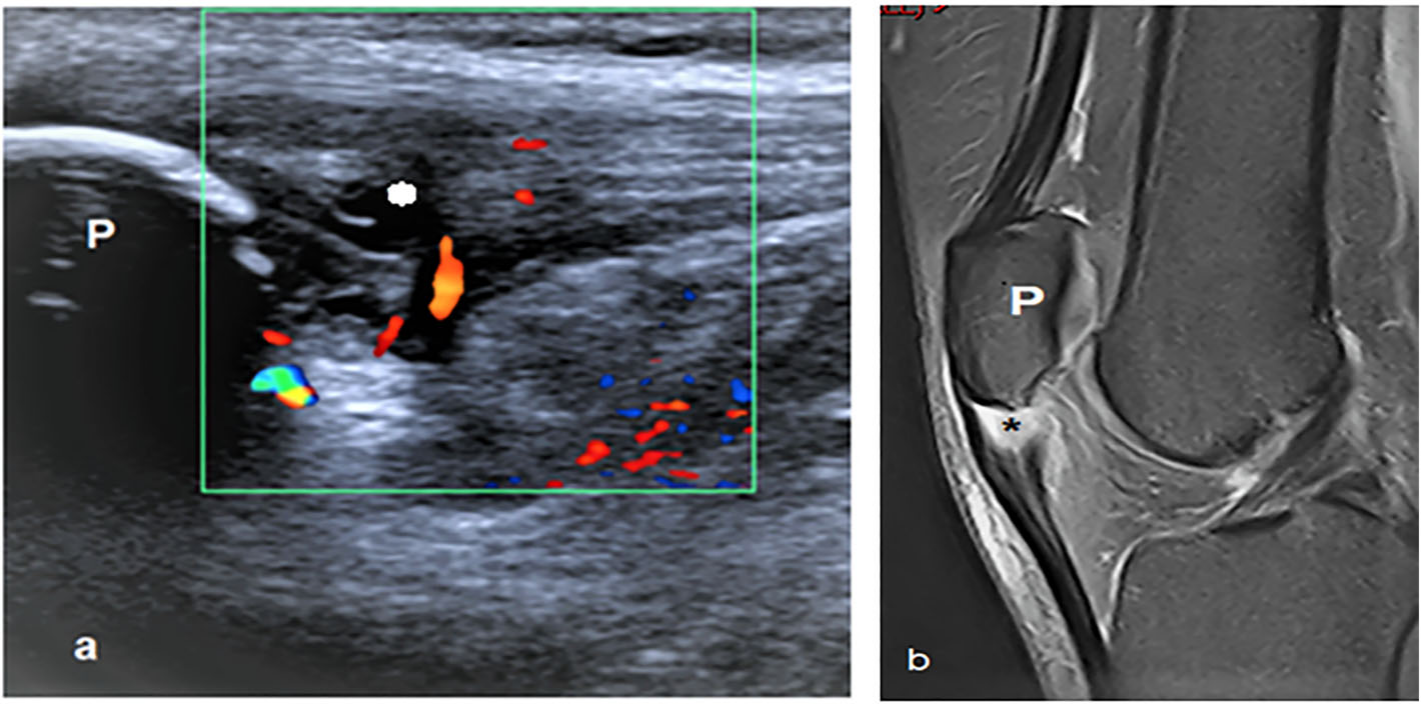
A 22-year-old male with left patellar tendinopathy. **a** Long axis ultrasound image demonstrates the thickened proximal part of the patellar tendon with cystic changes (*). Color Doppler mapping shows increased vascularity in and around the tendon. **b** Sagittal PDFS shows thickened proximal patellar tendon with fluid signal (*). P, patella

**Fig. 10:**
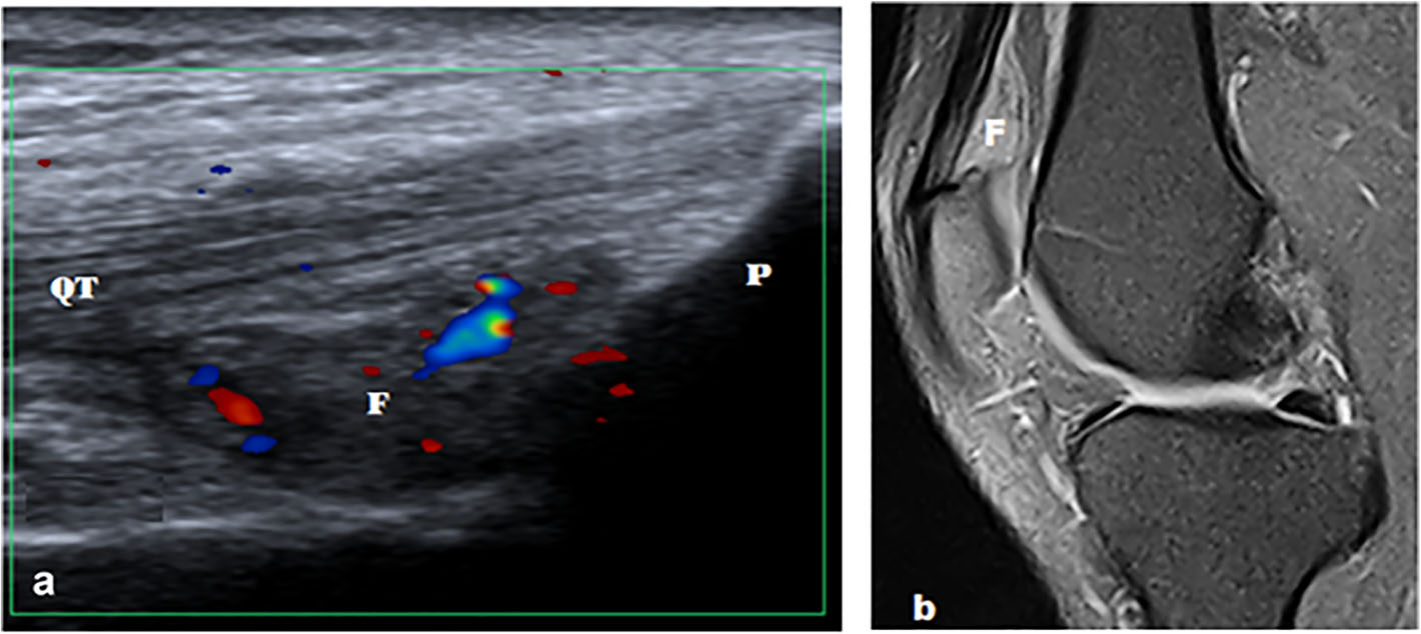
A 37-year-old male with left suprapatellar pad of fat impingement. **a** Long axis ultrasound image shows hypoechoic suprapatellar fat pad with convex posterior margin. Color Doppler ultrasound shows increased vascularity. **b** Sagittal PDFS MR image shows hyperintense suprapatellar fat pad with convex posterior margin. QT, quadriceps tendon; P, patella; F, suprapatellar fat pad

Representative cases of our study are shown in Figs. 5, 6, 7, 8, 9, and 10.

## Discussion

The current study highlighted the role of ultrasonography in the diagnosis of AKP. The overall results are encouraging and demonstrated the high diagnostic accuracy of ultrasonography (85.3% sensitivity, 100% specificity, 100% PPV, and 57.4% NPV) compared to MRI. Ultrasonography detected 195 out of 259 findings detected by MRI. Some of these findings could be the cause of the AKP like tendinopathy, fat impingement, and chondropathy, whereas others were just a sign of associated pathology like joint effusion and anterior subcutaneous edema, which may strengthen an equivocal or suspected clinical diagnosis. The ultrasonography detected most of the pathological findings in this study. The early and fast diagnosis by ultrasonography could allow the orthopedic to start the patient management plane without the usual delay for requesting and scheduling MRI examinations that would have a good impact on patient recovery.

According to the study done by Artul et al. [19], 34% of ultrasonography reports were negative, and 66% were positive. In the present study, 29% (45 knees) of ultrasonography reports and 16.8% (26 knees) of MRI reports were normal.

Lee and Chow [20] reported that ultrasonography is a sensitive tool to assess knee joint effusion, and a minimal amount of joint effusion as low as 7 to 10 ml could be optimally detected. According to the Draghi et al. [21] study, ultrasonography had 81.3% sensitivity and 100% specificity in detecting knee effusion. In the present study, ultrasonography showed a sensitivity of 91.5% and specificity of 95.8% in detecting knee effusion. Ultrasonography failed to detect five knees as the effusions were minimal and presented in front of the anterior cruciate ligament. On the other hand, possible improvement in the gap period between ultrasonography and MRI may be responsible for the false-positive results.

In the relevant literature, the detection of cartilage defects commonly refers to trochlear cartilage. The patellar cartilage is not routinely assessed by ultrasonography, as it is usually shaded by the patella. Cao et al. [22] stated that ultrasonography is considered a promising screening tool for assessment of trochlear cartilage defects as it showed a sensitivity range of 62.2 to 69.4% and specificity range of 90.5 to 92.9%. In the present study, ultrasonography showed a sensitivity of 71.9% and a specificity of 98.4% in detecting trochlear cartilage defects. In the 9 false-negative knees, the trochlear defects were overlooked by ultrasonography as they were deeply located in the intercondylar fossa. All patellar cartilage defects were overlooked by ultrasonography because they were obscured by the patellar shadow during the examination.

Unlu et al. [23] reported that anterior subcutaneous edema is a common finding (82.7%) on routine knee MRI and is significantly associated with old age, overweight, and patellofemoral chondral changes. In the present study, ultrasonography showed a sensitivity of 77.4% in detecting subcutaneous edema.

Knee’s synovial plica syndrome is a commonly overlooked cause of AKP [24]. Anatomically, the infrapatellar plica is the most common plica, followed by suprapatellar plica, and lastly, the medial patellar plica, which is considered the most symptomatic [25]. In this study, ultrasonography showed a sensitivity of 78.5% and specificity of 100% in detecting synovial plicae. The infrapatellar plica was the least common type of plica in our study, contrary to what was reported by Kheiralla [25], probably due to the type of our group, as they were symptomatic for AKP.

Patellar tendinopathy is a common cause of AKP, especially in athletes. It mainly affects the proximal aspect of the tendon. Ultrasonography had 87% sensitivity in detecting patellar tendinopathy [26, 27]. In this study, the sensitivity of ultrasonography was 84%.

To the best of our knowledge, detection of quadriceps tendon pathology has been reported while commonly referring to the quadriceps tendon tear in athletes, and most of the related literature focused only on MRI [25, 28]. In this study, ultrasonography showed 87.5% sensitivity, compared to 72.5% reported by King et al. [29].

Fat pad impingement syndromes most often affect the suprapatellar and superolateral Hoffa’s fat pads [30, 31]. These fat pads normally act to promote both joint lubrication and joint stability [32]. When impinged due to patellar maltracking, these fat pads display obscuration of normal fat and increased vascularity in these regions [33]. Gutierrez et al. [34] ensured the ability of ultrasonography in the detection of suprapatellar impingement. Tsavalas and Karantanas [35] reported a prevalence rate of 12% among patients with AKP. In our results, the prevalence rate of the suprapatellar fat impingement was 12.3%. Ultrasonography had 84.2% sensitivity and 100% specificity in detecting suprapatellar fat impingement. Color Doppler ultrasound showed increased vascularity in 3 knees due to concomitant inflammation.

Draghi et al. [21] reported that MRI is the modality of choice in the assessment of pathological changes of the Hoffa’s fat pad. Mikkilineni et al. [36] prospectively emphasized that ultrasonography may be valuable for the diagnosis of impingement of the Hoffa’s fat pad, which needs more research. In this study, the sensitivity and specificity of ultrasonography were 66.7% and 100%, respectively for diagnosing impingement of the Hoffa’s fat pad. To our knowledge, no results were published in the literature to compare.

According to Draghi et al. [37], ultrasonography had 100% sensitivity and specificity in detecting deep infrapatellar bursa. In the present study, the sensitivity and specificity of ultrasonography were 66.7% and 100%, respectively.

Our results matched Blankstein et al. [38] in the assessment of the bipartite patella and reported 100% sensitivity and specificity of ultrasonography.

Gel stand-off pad is an aqueous, flexible, easily available, disposable spacer, widely used in B-mode ultrasonography approach of superficial lesions or difficult-to-visualize areas. Moreover, it allows the detection of otherwise-missed peri- or intra-lesional flow signals on Doppler imaging [39]. In our study, we did not use the gel pad and used plenty of thick gel to avoid loss of contact. Therefore, further studies discussing the value of using gel stand-off pad in musculoskeletal ultrasonography are recommended.

Although ultrasonography had high diagnostic accuracy, a substantial number of lesions were missed. The main lesions missed by ultrasonography and detected by MRI were the patellar cartilage defects (ultrasonography missed all lesions) and the trochlear cartilage defects (ultrasonography missed 9 lesions). Accordingly, ultrasonography can be used in the diagnosis and screening of patients with AKP and can be used as an alternative to MRI when MRI was unavailable or contraindicated. MRI is indicated if a patellar cartilage defect is clinically suspected or the ultrasonography yielded negative results.

Our study has some limitations: First, there was a wide variety of findings; some of these findings may not be the actual cause of the AKP, and the others had no previously published results in the literature to compare. This generalizability may result in substantial compromises on the quality of findings. However, this study was an attempt to publish a comprehensive review about the diagnostic accuracy of ultrasonography in patients with AKP. Second, there was higher sensitivity of ultrasonography in detecting joint effusion, which was at the same time the most common finding in the current study. The perfection of ultrasonography in detecting joint effusion increased the overall sensitivity of ultrasonography in the detection of pathological findings in the AKP patients and masked its weakness in detecting other entities like patellar cartilage defect and infrapatellar plica. Third, unfortunately, all the ultrasonography examinations were performed by one radiologist. Hence, there was no chance to make the intra- and inter-observer agreement. Further studies discussing the possible variability of ultrasonographic signs among radiologists and providing data for operator intra- and inter-observer agreement are recommended. Fourth, the patients included in this study were already scheduled for an MRI examination, which could produce a selection bias. Nevertheless, not all patients scheduled for an MRI examination in this study were severed or complicated. Finally, lack of surgical data and clinical follow-up.

## Conclusion

Ultrasonography can be used to diagnose patients with AKP; it showed high diagnostic accuracy in detecting most of the findings. Although MRI is the gold standard technique for AKP imaging, ultrasonography can be used to make a swift screening and assessment of the painful anterior knee and can be used as an alternative to MRI when MRI is unavailable or contraindicated. MRI may be warranted if a patellar cartilage defect is clinically suspected or the ultrasonography yielded negative results.

## Data Availability

The datasets used and/or analyzed during the current study are available from the corresponding author on reasonable request.

## Abbreviations

AKP: Anterior knee pain
AUC: Area under the curve
MRI: Magnetic resonance imaging
ROC: Receiver operating characteristic curve
